# Ice I_c_ without stacking disorder by evacuating hydrogen from hydrogen hydrate

**DOI:** 10.1038/s41467-020-14346-5

**Published:** 2020-02-03

**Authors:** Kazuki Komatsu, Shinichi Machida, Fumiya Noritake, Takanori Hattori, Asami Sano-Furukawa, Ryo Yamane, Keishiro Yamashita, Hiroyuki Kagi

**Affiliations:** 1 Geochemical Research Center, Graduate School of Science, The University of Tokyo, 7-3-1 Hongo, Bunkyo-ku, Tokyo 113-0033 Japan; 2 Neutron Science and Technology Center, CROSS, 162-1 Shirakata, Tokai, Naka, Ibaraki 319-1106 Japan; 3 Graduate Faculty of Interdisciplinary Research, University of Yamanashi, 4-3-11 Takeda, Kofu, Yamanashi 400-8511 Japan; 4 Computational Engineering Applications Unit, RIKEN, 2-1 Hirosawa, Wako, Saitama 351-0198 Japan; 5 J-PARC Center, Japan Atomic Energy Agency, 2-4 Shirakata, Tokai, Naka, Ibaraki 319-1195 Japan

**Keywords:** Chemical physics, Phase transitions and critical phenomena

## Abstract

Water freezes below 0 °C at ambient pressure ordinarily to ice I_h_, with hexagonal stacking sequence. Under certain conditions, ice with a cubic stacking sequence can also be formed, but ideal ice I_c_ without stacking-disorder has never been formed until recently. Here we demonstrate a route to obtain ice I_c_ without stacking-disorder by degassing hydrogen from the high-pressure form of hydrogen hydrate, C_2_, which has a host framework isostructural with ice I_c_. The stacking-disorder free ice I_c_ is formed from C_2_ via an intermediate amorphous or nano-crystalline form under decompression, unlike the direct transformations occurring in ice XVI from neon hydrate, or ice XVII from hydrogen hydrate. The obtained ice I_c_ shows remarkable thermal stability, until the phase transition to ice I_h_ at 250 K, originating from the lack of dislocations. This discovery of ideal ice I_c_ will promote understanding of the role of stacking-disorder on the physical properties of ice as a counter end-member of ice I_h_.

## Abstract (web summary)

Metastable cubic ice has been identified in several conditions relevant to geo and astrochemistry, but was always characterized by stacking disorder. Here the authors synthesize a hydrogen hydrate and degas hydrogen, obtaining pure non-defected cubic ice, observed by X-ray and neutron diffraction.

## Introduction

Water freezes below 0 °C at ambient pressure, ordinarily to ice I_h_ with a hexagonal stacking sequence. However, it is also known to produce “ice I_c_” nominally with a cubic stacking sequence under certain conditions^1^, and its existence in Earth’s atmosphere^2–4^, or in comets^5,6^ is debated. “Ice I_c_”, or called as cubic ice, was first identified in 1943 by König^7^, who used electron microscopy to study the condensation of ice from water vapor to a cold substrate. Subsequently, many different routes to “ice I_c_” have been established, such as the dissociation of gas hydrates, warming amorphous ices or annealing high-pressure ices recovered at ambient pressure, freezing of μ- or nano-confined water (see ref. ^1^). Despite the numerous studies on “ice I_c_”, its structure has not been fully verified, because the diffraction patterns of “ice I_c_” show signatures of stacking disorder^1,8,9^, and ideal ice I_c_ without stacking disorder had not been formed until very recently^10^. This is the reason why “ice I_c_” is double-quoted^1^, and it is recently proposed that the stacking-disordered ice should not be termed as ice I_c_, but as ice I_sd_^8^.

“Ice I_c_” (ice I_sd_) is known as a metastable form of ice at atmospheric pressure. But, recent computer simulations suggest that even ice I_sd_ could be the stable phase for crystallites up to sizes of at least 100,000 molecules^11^. The stability of stacking-disordered ices is extremely important because of the ubiquitous nature of ice. Stacking-disordered ice can be characterized by the degree of ice cubicity, *χ*, which is defined as the fraction of cubic stacking^1,8,9,12,13^. Until very recently, the highest cubicity was limited to ~80%^8,14^, but it has been reported that ideal ice I_c_ with 100% cubicity has been obtained by annealing ice XVII^10^.

Recently new ice polymorphs, ice XVI^15^ and ice XVII^16,17^ are obtained by degassing gas molecules from neon and hydrogen hydrates, respectively. From these findings, we hypothesized that ideal ice I_c_ could be obtained by degassing hydrogen from hydrogen hydrate, C_2_. Five different phases in the H_2_–H_2_O system have been reported to date (see ref. ^18^): Among them, neutron diffraction experiments have never been conducted for the higher-pressure phases, C_1_ and C_2_, probably due to the technical difficulty in loading hydrogen into a pressure vessel, or compressing it to pressures in the giga-pascal range. To synthesize ideal ice I_c_, decompression under low-temperature conditions for degassing is necessary, which is also not straight-forward using conventional pressure-temperature controlling systems. We have developed a Mito system^19^, and have overcome these technical difficulties (see details in Methods).

Here we present the neutron and X-ray diffraction results showing ice I_c_ without stacking disorder, obtained from degassing hydrogen hydrate C_2_. We also report an unexpected amorphous-like state in the transformation from C_2_ to ice I_c_, and the thermal stability of ice I_c_.

## Results

### The route to obtain ice I_c_

**Fig. 1:**
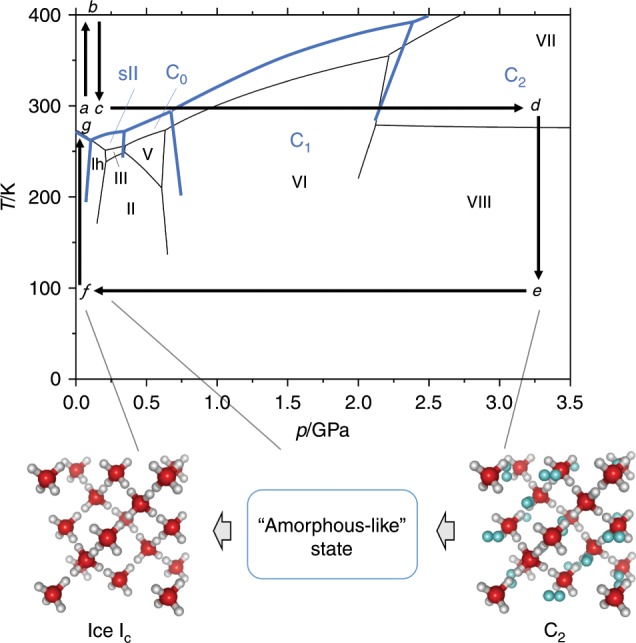
Phase diagram of hydrogen hydrate and ice with experimental paths in this study. Phase boundaries for hydrogen hydrates and ices are drawn using thick blue lines and thin black lines, respectively. Experimental *p*-*T* paths are shown as black arrows in alphabetical sequence from *a* to *g*. The structural models for a high-pressure form of hydrogen hydrate, C_2_, and ice I_c_ are schematically drawn with a newly found amorphous-like state as an intermediate transitional state from C_2_ to ice I_c_. Red, white, and light blue balls in the structure model depict oxygen, hydrogen in water molecules, and hydrogen in guest molecules, respectively. Note that hydrogens in water molecules are disordered, so that two of four possible sites surrounding one oxygen are actually occupied.

We started by using a mixture of D_2_O and MgD_2_, which is an internal deuterium source, to synthesize hydrogen hydrate, C_2_. After loading the mixture into a pressure-temperature controlling system, MgD_2_ was decomposed by heating at 403 K and at ca. 0 GPa for 1 h through a nominal reaction of MgD_2_ + 3D_2_O → Mg(OD)_2_ + 2D_2_ + D_2_O (at *b* in Fig. 1, the observed neutron diffraction patterns are shown in Supplementary Fig. 1). Then, the samples were cooled to room temperature (at *c* in Fig. 1) and typically compressed up to ~3 GPa until a C_2_ phase was observed (at *d* in Fig. 1).

**Fig. 2:**
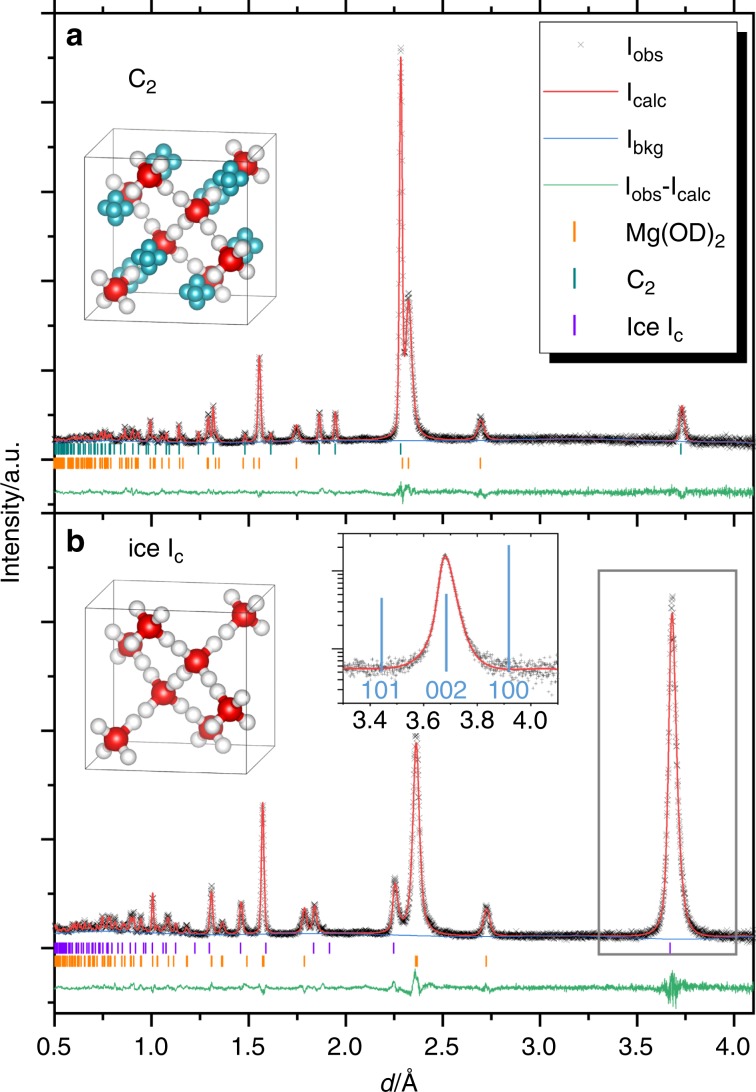
Results of Rietveld analyses for neutron diffraction patterns. The patterns of **a** hydrogen hydrate, C_2_, and **b** ice I_c_ were obtained at 3.3 GPa and 300 K (at *d* in Fig. 1), and at 0 GPa and 130 K (in the path *f* → *g*). The inset diffraction pattern in **b** shows the expanded area for 111 reflections, shown as a box in the main figure, with logarithmic scale. The calculated peak positions of ice I_h_ are also shown as light blue lines with their indices in the inset. Structure models for C_2_ and ice I_c_ are also shown as insets in **a** and **b**, respectively.

The neutron diffraction pattern for the C_2_ phase obtained at 3.3 GPa and 300 K (at *d* in Fig. 1) was analyzed by the Rietveld method. We adopted a splitting site model for guest D atoms located at the 48*f* site (*x*, 1/8, 1/8), and the host structure was identical to ice I_c_^9^ ($Fd\,{\overline{3}}m$, O at the 8*b* site (3/8, 3/8, 3/8), D at the 32*e* site (*x*, *x*, *x*)). The calculated diffraction pattern was in good agreement with the observed one, as shown in Fig. 2a. The refined structural parameters are listed in Supplementary Table 1.

The sample was then cooled from 300 K to 100 K at around 3 GPa (path *d* → *e*). In the diffraction pattern taken at *e* in Fig. 1, peaks from solid deuterium (phase I) appeared at around 200 K (Supplementary Fig. 2), which is consistent with the known melting curve of hydrogen^20^. This observation indicates that fluid deuterium coexisted with C_2_ through the path from *b* to *d*.

**Fig. 3:**
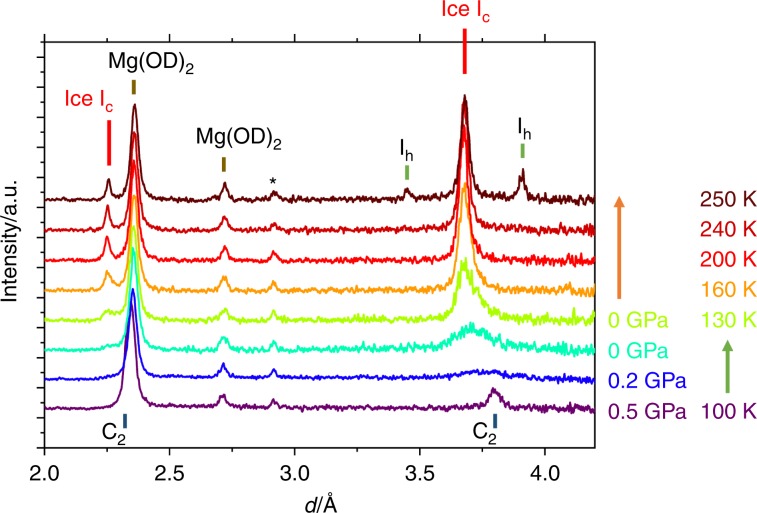
Neutron diffraction patterns showing the transformation from C_2_ to ice I_c_. The patterns were obtained with decreasing pressure at 100 K (path *e* → *f*) and with increasing temperature at 0 GPa (path *f* → *g*). Corresponding temperatures and pressures are shown at the right side of the respective patterns, and the arrows mean that temperature or pressure kept constant. Most observed peaks are identified as C_2_, ice I_c_, Mg(OD)_2_, or ice I_h_. The peak marked by an asterisk is a parasitic peak from the high-pressure cell.

The C_2_ phase persisted at pressures at least as low as 0.5 GPa on decompression at 100 K (path *e* → *f*). However, surprisingly, the Bragg peaks of C_2_ mostly disappeared at 0.2 GPa (Fig. 3). This phenomenon is totally unexpected, because the host structure of gas hydrates retains its framework in the previous cases with ice XVI^15^ and XVII^16^. The sample was further decompressed to 0 GPa and evacuated using a turbo-molecular-pump. The broad peaks corresponding to ice I_c_ appeared at this stage. The peak disappearance of C_2_ before the appearance of ice I_c_ was reproducibly observed in at least two separate neutron runs and one X-ray diffraction run for a hydrogenated sample (Supplementary Fig. 3). In the neutron diffraction pattern at 0.2 GPa, except for the Bragg peaks from Mg(OD)_2_, only a broad peak was observed at around *d* = 3.75 Å, which was between the peak positions of 111 of C_2_ and that of Ice I_c_ (Fig. 3). This fact implies that this state does not have long-range periodicity like a normal crystal, but has only local-ordering like an amorphous or nano-crystal. Considering the observed *d*-spacing, this amorphous-like form would be an intermediate transition state from C_2_ to ice I_c_, which forms while hydrogen molecules are partially degassed. It is highly likely that this apparent amorphization is derived from the lattice mismatch between C_2_ and ice I_c_, originating from the relatively small cage in the host framework of the ice I_c_ structure.

From the X-ray diffraction run, ice I_c_, which may partially include molecular hydrogen, even appeared at 0.1 GPa through the transition from the C_2_ phase to the amorphous-like state, even under pressure (Supplementary Fig. 3). This also represents a difference from the previous cases of ice XVI and XVII; ice XVI is formed under evacuation^15^, and hydrogen molecules can be refilled into ice XVII at an order of 10 bar of pressure^16^. It is worth noting that the partially degassed states are allowed in the cases of both ice XVI and XVII, so that the guest molecules can be continuously degassed from a fully occupied state to an empty state. The observed phase-separation behavior even under pressure in the ice H_2_-H_2_O system indicates that the partially degassed C_2_ phase would be unstable compared to the fully occupied or emptied phases, probably due to their lattice-mismatch.

The Bragg peaks in the neutron diffraction pattern for ice I_c_ obtained at 100 K were still broad, probably due to the small crystallite size and/or the remaining guest hydrogen molecules. The peaks of ice I_c_ sharpened with increasing temperature. This sharpening is dependent not only on temperature but also on time, which indicates that it is kinetic behavior.

### Structure refinement for ice I_c_

We conducted a separate run in order to obtain a neutron diffraction pattern for the structure refinement of the ice I_c_. In this run, the neutron diffraction pattern was obtained at 130 K, which is well below the temperature at which the nucleation of ice I_h_ occurs^21^. We confirmed that the peak width did not change in the temperature region from 130 K to 180 K, such that the peak sharpening was almost complete, even at 130 K. The obtained neutron diffraction pattern was well fitted using the ice I_c_ structure model^9^, as shown in Fig. 2b and Supplementary Table 1. We also conducted the Rietveld analysis using C_2_ structure model, and found that the occupancy of the D2 site is zero, within experimental error (occ(D2) = −0.001(1)). This shows that the guest hydrogen molecules are below the detectable limit at 130 K under evacuation. The peak profile around 111 peak of ice I_c_ has neither the feature of stacking disorder nor the peaks from ice I_h_, as shown in the diffraction pattern in the region at around *d* = 3.9 Å, where the strongest 100 reflection of ice I_h_ is expected (see inset in Fig. 2b, and more detailed discussion for the peak broadening for ice I_c_ is described in Supplementary Note, Supplementary Table 2 and Supplementary Fig. 4). This should be a clear indication of the presence of ideal ice I_c_ without stacking disorder (*χ* = 100%)^13^, as clear as the recent discovery of ideal ice I_c_ by annealing ice XVII^10^.

### Thermal stability of ice I_c_

It is also noteworthy that the ice I_c_ surprisingly persists up to at least 240 K until ice I_h_ started to appear at 250 K (Fig. 3). The temperature of 240 K corresponds to the upper limit of the reported metastable region of “ice I_c_” (ice I_sd_)^1^. However, in stacking-disordered ice, the cubic stacking sequence starts to change into a hexagonal stacking sequence at a much lower temperature, and the phase transition to ice I_h_ is completed at 240 K. The notable stability of the ice I_c_ would be derived from the lack of stacking disorder. The stacking-disordered ice has more dislocations, which promote the phase transformation from ice I_sd_ to ice I_h_ by reducing the activation energy required to change the stacking sequence^22^. This is also supported by a recent mesoscopic-size calculation^23^. Note here that the critical temperature of 240 K has been identified as the temperature above which ice I_h_ without cubic stacking faults forms spontaneously, which is the reason for the anomalous self-preservation regime of natural gas hydrates^24^.

The diffraction pattern observed at 250 K looks a mixture of bulk ice I_c_ and I_h_, rather than stacking-disordered ice with many stacking faults, judging from “stackogram” reported in the literatures^8,13^. At 250 K, crystal growth would be dominant, rather than crystal nucleation. Therefore, once a crystallite nucleates, it quickly grows before other crystallites nucleate, resulting in the mixture of ice I_c_ and I_h_, rather than stacking-disordered ice. This observation also suggests a smaller number of dislocations in the ice I_c_ observed in this study. On the contrary, the remarkable stability of the ice I_c_ and the bulk mixture of ice I_c_ and I_h_ at 250 K strongly support the conclusion that the obtained ice is not stacking-disordered, and it can therefore be called ice I_c_ without the need for quotation marks.

The discovery of ideal ice I_c_ will allow us to research the real physical properties of ice I_c_ without stacking disorder. For example, accurate heat capacity or vapor pressure measurements from low temperature will provide the free energy of ice I_c_, which settle the long-standing argument for the thermodynamic stability of ice I_c_ compared to ice I_h_. The physical properties of the ideal ice I_c_ are also important to understand how stacking disorder plays a role in the physical properties of ice I_sd_. For instance, since the thermal conductivity of ice I_sd_ is significantly smaller than ice I_h_^25^, the difference of thermal conductivities between ices I_h_ and I_c_ will emboss the effect of stacking disorder. It is also interesting what will be happened when ice I_c_ is compressed under low temperature; whichever ice I_c_ will be transformed into HDA (High Density Amorphous ice^26^) or not? In any case, it is worth conducting what we previously did for ice I_h_, to this ideal ice I_c_ as well.

## Methods

### Synthesis of MgD_2_

MgD_2_, used as the starting material in this study, was synthesized from reagent-grade MgH_2_ as follows. MgH_2_ powder (Wako pure chemical industries, Ltd.) was purchased and further ground in an agate motor to increase the surface area, after which it was placed in a copper tube with a diameter of 4 mm and a length of 40 mm. The tube was mechanically sealed and but not welded, allowing the transfer of hydrogen gas. The copper tube was inserted into a 1/4” Inconel tube and connected in parallel to a deuterium gas cylinder and a turbo-molecular pump (TMP) with 1/16” stainless tubes and stop bulbs. The Inconel tube, including the sample copper tube, was heated to 773 K for 1 h using a tube furnace under evacuation using the TMP. Under these conditions, MgH_2_ completely decomposed to Mg and H_2_^27^, and the degassed H_2_ was evacuated. Then, the D_2_ gas was introduced up to 4 MPa, and the temperature was cycled at the rate of 1 K/min between 673 K and 773 K while keeping the pressure at 4 MPa, which represents stable and unstable conditions for MgH_2_^27^, and this temperature cycle was repeated for 20 times. This activation process is necessary for the reaction Mg + D_2_ → MgD_2_. Finally, the *p*-*T* conditions were maintained at 673 K and 4 MPa for 3 days to complete the reaction. The recovered sample was analyzed by powder X-ray diffraction (MiniFlex-II, Rigaku) and identified to be MgD_2_ with a trace amount of MgO. Both MgD_2_ and MgO react with D_2_O and produce Mg(OD)_2_, so this small amount of MgO does not affect the conclusion of this study.

### Neutron diffraction and *p*-*T* control

Neutron powder diffraction experiments were conducted at the beamline PLANET^28^ in the Material and Life Science Experiment Facility (MLF) of J-PARC, Ibaraki, Japan. The incident beam consists of 25 Hz pulsed spallation neutrons produced from a liquid Hg target via a decoupled moderator and traveled through collimators, choppers and supermirror guides to the sample positioned at 25.0 m from the moderator^28^. Approx. 20 mg of MgD_2_, synthesized as described above, was filled into TiZr null scattering gaskets, and D_2_O water (99.9%, Wako pure chemical industries, Ltd.) was dropped on the MgD_2_ powder, resulting in the molar ratio of MgD_2_:D_2_O~1:3. The gaskets were sandwiched between a pair of tungsten carbide anvils, and loaded by using a hybrid Mito system, which is a modified version of an original pressure-temperature variable Mito system^19^. The hybrid Mito system uses both flowing liquid nitrogen and a 4 K cryostat (RDK-415D, Sumitomo Heavy Industries, Ltd.), which allows us to control temperature rapidly, owing to the large latent heat of liquid nitrogen and efficient thermal insulation by zirconia and GFRP seats. The hybrid Mito system also allows us to achieve temperatures below 77 K, and reach a minimum temperature of ~35 K, owing to the cryostat. Another remarkable feature of the hybrid Mito system is that it affords pressure control, even at low temperature, as well as the original Mito system, which is indispensable for this study. Flexible copper cloths were attached on the support rings of the anvils, and the cloths were placed in contact with the cold head of the cryostat for thermal conduction. The accessible minimum temperature of the hybrid Mito system is ~35 K, which may be the current technical limitation due to an unavoidable influx of heat from the surrounding cell. The sample pressure was estimated from the observed lattice parameter of Mg(OD)_2_ brucite using the equation of states^29^ and the observed unit cell volume of brucite at 0 GPa, assuming the temperature derivative of the bulk modulus of brucite, *dK*/*dT*, was ~0. Although this assumption may cause some error in the pressure estimated at low temperature, we placed emphasis on avoiding unwanted Bragg peaks from additional sources of pressure marker. Moreover, the error would be too small to affect the conclusion. The sample position was aligned by scanning to maximize the sample scattering intensity. The obtained intensities from the sample in the cell was subtracted by the intensity of the empty cell, and subsequently normalized by the attenuation corrected intensity of vanadium pellet in the cell, which was also subtracted by the intensity of the empty cell^30^. The Rietveld analyses were performed using the GSAS^31^ with EXPGUI^32^, and the crystal structure was drawn with the VESTA program^33^. The GSAS TOF profile function 3^31^ is used as the profile function in the Rietveld analyses.

### X-ray diffraction

Powder X-ray diffraction measurements using a H_2_O (Milli-Q) and MgH_2_ (Wako pure chemical industries, Ltd.) mixture as starting materials were performed at the beamline BL-18C in the Photon Factory (KEK, Tsukuba, Japan). Samples were exposed to 0.6134 Å monochromatized synchrotron radiation, and the diffracted scattering was detected by an imaging plate (IP). The pressure was generated using CuBe alloy diamond-anvil cells and the temperature was controlled using a 4 K GM cryostat (MiniStat, Iwatani Co.) equipped with a temperature controller (Model 335, Lakeshore). Sample pressure was estimated from the difference in the R1 line wavelengths of rubies inside and outside the sample chamber^34^. The temperature was monitored using a Si-diode sensor inserted in the cold head edge. We confirmed that the measured temperature was almost the same as that of the diamond anvils after temperature stabilization. The experimental *p*-*T* path was basically identical to the case of neutron diffraction, as shown in Fig. 1, while the achieved pressure at path *d* was 4.1 GPa.

One conically shaped Boehler-Almax type diamond-anvil^35^ with a 0.6 mm culet was placed in the direction of the detector with an opening angle of 2*θ* < 40°, whereas a conventional anvil with a 0.8 mm culet was positioned in the direction of the X-ray source. A CuBe plate with a hole of diameter 0.3 mm and an initial thickness of 0.2 mm was used as a gasket. This gasket was not subjected to pre-indentation. The load was applied by driving the piston by bellows using a He gas cylinder. The bellows allow us to control pressure at a few kbar more precisely than conventionally used membranes.

### DFT calculations

Quantum Espresso^36^ was used for the DFT calculations^37,38^. We used Perdew-Burke-Ernzerhof (so-called PBE) type nonempirical exchange-correlation functions^39^ for this study. The pseudopotentials were derived using projector augmented-wave approximation^40^. The dispersion effects were taken into account using the exchange-hole dipole moment method (XDM), which calculates coefficients for polynomial of DFT-D dispersion energy^41^ from the exchange-hole dipole moment calculated from simulated electron wave function^42,43^. XDM damping function parameters are taken from^44^. The enthalpies of four possible configurations for the ordered form of ice I_c_ were calculated within a unit cell with a kinetic energy cutoff of 70 Ry and a Brillouin zone k mesh of 8 × 8 × 8. The cell parameters and atomic coordinates were optimized using BFGS quasi-Newtonian methods at atmospheric pressure.

## Supplementary information

Supplementary Information

## Data Availability

The primary data that support the plots within this paper and other finding of this study are available from the corresponding author on reasonable request. The neutron crystallographic coordinates for structures reported in this study have been deposited at the Cambridge Crystallographic Data Centre (CCDC), under deposition numbers 1973757, 1973759. These data can be obtained free of charge from The Cambridge Crystallographic Data Centre via www.ccdc.cam.ac.uk/data_request/cif.
