# Supplementary material for: Ice Ic without stacking disorder by evacuating hydrogen from hydrogen hydrate
Source: Nat Commun. 2020 Feb 3;11:464. doi: 10.1038/s41467-020-14346-5 (PMC6997176; doi:10.1038/s41467-020-14346-5)
Supplement: Supplementary file 1 — Supplementary Information [file 41467_2020_14346_MOESM1_ESM.docx]

Supplementary Information

**Ice I_c_ without stacking disorder
by evacuating hydrogen from hydrogen hydrate**

Kazuki Komatsu^1*^, Shinichi Machida^2^, Fumiya Noritake^3,4^, Takanori Hattori^5^, Asami Sano-Furukawa^5^, Ryo Yamane^1^, Keishiro Yamashita^1^ & Hiroyuki Kagi^1^

^1^Geochemical Research Center, Graduate School of Science, The University of Tokyo, 7-3-1 Hongo, Bunkyo-ku, Tokyo 113-0033, Japan

^2^Neutron Science and Technology Center, CROSS, 162-1 Shirakata, Tokai, Naka, Ibaraki 319-1106, Japan

^3^Graduate Faculty of Interdisciplinary Research, University of Yamanashi, 4-3-11 Takeda, Kofu, Yamanashi 400-8511, Japan

^4^Computational Engineering Applications Unit, RIKEN, 2-1 Hirosawa, Wako, Saitama 351-0198, Japan

^5^J-PARC Center, Japan Atomic Energy Agency, 2-4 Shirakata, Tokai, Naka, Ibaraki 319-1195, Japan

*Corresponding author

e-mail: kom@eqchem.s.u-tokyo.ac.jp

**Supplementary Table 1 | Experimental details and crystallographic data for C_2_ and ice Ic.**

|  | C_2_ | Ice Ic | |
| --- | --- | --- | --- |
| Chemical formula | D_2_·D_2_O | D_2_O | |
| Molecular weight | 40.055 | 20.028 | |
| Crystal system | Cubic | Cubic | |
| Space group | *Fdm* | *Fdm* | |
| Temperature (K) | 300 | 100 | |
| Pressure (GPa) | 3.3 | 0 | |
| *a* (Å) | 6.45307(8) | 6.3560(2) | |
| *V* (Å^3^) | 268.719(11) | 256.77(3) | |
| *Z* | 8 | 8 | |
| Radiation type | Spallation neutron | | Spallation neutron |
| Diffractometer | PLANET (BL11), MLF, J-PARC | | PLANET (BL11), MLF, J-PARC |
| Specimen mounting | Pressure-temperature controlling system (the hybrid MITO system) | | Pressure-temperature controlling system (the hybrid MITO system) |
| *R*_p_ | 0.0379 | 0.0532 | |
| *R*_wp_ | 0.0425 | 0.0605 | |
| *R*(*F*^2^) | 0.114 | 0.0772 | |
| *χ*^2^ | 1.975 | 2.556 | |
| No. of data points | 3525 | 3525 | |
| No. of parameters | 29 | 24 | |
|  |  |  | |
| *U*_iso_(O) | 0.0113(7) | 0.0181(7) | |
| *x*(D1^*^) | 0.4607(2) | 0.4675(3) | |
| occ(D1^*^) | 0.5 (fixed) | 0.5 (fixed) | |
| *U*_iso_(D1^*^) | 0.0250(9) | 0.0239(7) | |
| *x*(D2^**^) | 0.0636(5) | - | |
| occ(D2^**^) | 0.310(1) | - | |
| *U*_iso_(D2^**^) | 0.0250(9)  (=*U*_iso_(D1)) | - | |

^*^D1 belongs to a water molecule in the host structure.

^**^D2 belongs to the guest deuterium molecule. *U*_iso_(D2) is constrained to be the same value as *U*_iso_(D1), because of the severe correlation between atomic coordinates and occupancies.

**Supplementary Figure 1 | Neutron diffraction patterns showing decomposition of MgD_2_.** The bottom pattern shows starting materials taken at 300 K (at *a* in Fig. 1), and the above shows time-resolved patterns at 403 K (at *b* in Fig. 1) for each 10 min. The obtained Bragg peaks are indexed as MgD_2_ (blue tick marks) or Mg(OD)_2_ (green tick marks). A broad peak at around *d* ~ 3 Å in the pattern at 300 K originated from liquid D_2_O, and the broad peak shifted to lower *d*-spacing at 403 K, which would show the existence of fluid D_2_ in the sample chamber.

**Supplementary Figure 2 | Sequential neutron diffraction patterns with decreasing temperature.** The patterns are taken at path *d*→*e*. Two 011 and 010 peaks from solid D_2_ (Phase I) are clearly seen, as shown by black triangles.

**Supplementary Figure 3 | X-ray diffraction patterns at 100 K with decreasing pressure.** (a) Whole patterns taken at path *e*→*f* and (b) an enlarged 2*θ* region from 8.5° to 10°, corresponding to the region shown in the black box in the figure on the left. Thick blue, green, and gray lines in the figure on the right schematically show the peak positions of 111 of ice Ic and C_2_, and 101 of ice VIII, respectively. Because the MgH_2_:H_2_O ratio in the starting material of this x-ray diffraction run was not sufficiently high to make pure C_2_, ice VIII remained in this run. At 0.50 GPa, 111 peaks of both C_2_ and ice Ic mostly disappeared, indicating the intermediate amorphous-like state at that pressure. The asterisk in the left figure denotes a scattering from Mylar® (polyester) film, which is used for a window material of the vacuum chamber.

**Supplementary Note**

**On the peak broadening of ice I_c_**

The peak width of the ice I_c_ obtained in this study (*Δd*/*d* ~ 1.2%) are significantly larger than the instrument resolution (*Δd*/*d* ~ 0.6%)^1^, as shown in Supplementary Fig. 4. This broadening cannot be the result of stacking-disorder, because the profile changes caused by the stacking-disorder are not simply broadening, but more complicated^2,3^. Considering the result that the peak width did not change up to 240 K once the peak sharpening converged, it is unlikely that the peak broadening of ice I_c_ could originate only from small crystalline size. Other broadening factors such as microstrain induced by lattice distortions should be taken into account (see also Supplementary Fig. 4). We assume the lattice distortion could be caused by hydrogen ordering, and then conducted the DFT calculations for the ordered form of ice I_c_ in order to estimate the degree of lattice distortion. There are four possible symmetrically non-equivalent configurations for ice I_c_ with unit cell volumes identical to that of the disordered ice I_c_, having the space groups of *Pna*2_1_, *I*4_1_*md*, *P*4_1,_ and *P*4_1_2_1_2^4^. Note that more number of configurations can be considerable for super cells of ice I_c_, but they are not regarded here. The optimized unit cell parameters show deviations from the cubic lattice (Supplementary Table 2). The deviation, which can be defined as the ratio of cell parameters between the characteristic axis (= the most deviated axis from cubic symmetry) and the other axes, ranges from 0.12 % for the *P*4_1_ model to 1.16 % for the *P*4_1_2_1_2 model. The degree of deviation is of an order similar to the peak broadening of 0.6% (1.2 % − 0.6 %) added to the instrument resolution. Although the obtained ice I_c_ does not have the long-range ordering for hydrogens, short-range ordering could result in the lattice distortion, which contributes to the peak broadening.

**Supplementary Table 2 | Results of DFT calculations.**

| Order model | 1 | 2 | 3 | 4 |
| --- | --- | --- | --- | --- |
| Crystal system | Orthorhombic^*^ | Tetragonal | Tetragonal | Tetragonal |
| Space Group | *Pna*2_1_^*^ | *I*4_1_*md* | *P*4_1_ | *P*4_1_2_1_2 |
| Dipole Moment (Debye) | 5.64 | 7.96 | 3.95 | 7.65E-05 |
| *a* | 6.1953 | 6.1711 | 6.1806 | 6.1352 |
| *b* | 6.1953 | 6.2007 | 6.1878 | 6.2070 |
| *c* | 6.1547 | 6.1711 | 6.1806 | 6.2070 |
| *α* | 90.0000 | 90.0000 | 90.0000 | 90.0000 |
| *β* | 90.0000 | 90.0000 | 90.0000 | 90.0000 |
| *γ* | 90.0242 | 90.0000 | 90.0000 | 90.0000 |
| Charac. axis /Cubic axis^**^ | 0.9935 | 1.0048 | 1.0012 | 0.9884 |
| *H*(eV)/8H_2_O | -4799.3535 | -4799.3755 | -4799.3358 | -4799.3249 |
| *ΔH*(eV)/8H_2_O | 0.0219281 | 0 | 0.0396700 | 0.0505637 |
| ^*^DFT calculation was started from the orthorhombic *Pna*2_1_ structure, but the symmetry of the optimized structure could be reduced from orthorhombic to monoclinic.  ^**^Ratio of cell parameters between the characteristic axis and cubic axis. The characteristic axis shows the greatest deviation from the cubic axis, as shown by shades. | | | | |

**Supplementary Figure 4 | Full width at half maximum divided by *d*-spacing (Δ*d/d*) as a function of *d*-spacing for C_2_ and ice Ic.** The error bars are ignored, as they are smaller than the size of symbols.

Supplementary References

1. Hattori, T. *et al.* Design and performance of high-pressure PLANET beamline at pulsed neutron source at J-PARC. *Nucl. Instr., Meth. Phys. Res. A* **780**, 55-67 (2015).

2. Hansen, T. C., Sippel, C. & Kuhs, W. F. Approximations to the full description of stacking disorder in ice I for powder diffraction. *Zeitschrift für Kristallographie* **230**, 75-86 (2015).

3. Malkin, T. L. *et al.* Stacking disorder in ice I. *Phys. Chem. Chem. Phys.* **17**, 60-76 (2015).

4. Geiger, P. *et al.* Proton Ordering of Cubic Ice Ic: Spectroscopy and Computer Simulations. *J. Phys. Chem. C* **118**, 10989-10997 (2014).
